# Lipid, adipokine and ghrelin levels in myocardial infarction patients with insulin resistance

**DOI:** 10.1186/1471-2261-14-7

**Published:** 2014-01-16

**Authors:** Olga Gruzdeva, Evgenya Uchasova, Ekaterina Belik, Yulia Dyleva, Ekaterina Shurygina, Olga Barbarash

**Affiliations:** 1 Research Institute for Complex Issues of Cardiovascular Diseases under the Siberian Branch of the Russian Academy of Medical Sciences, Kemerovo, Russia

**Keywords:** Insulin resistance, Myocardial infarction, Free fatty acids, Adipokines, Ghrelin

## Abstract

**Background:**

Insulin resistance (IR) is a risk factor for ischaemic heart disease and myocardial infarction (MI). IR often manifests in MI and is regarded as an independent predictor of in-hospital mortality, which can provide early risk stratification for recurrent acute coronary events.

**Methods:**

The study enrolled 200 patients (130 males and 70 females aged 61.4 ± 1.12 years) diagnosed with ST elevation MI. At days 1 and 12 from the MI onset, IR levels and lipid profiles, as well as serum glucose, insulin, adipokine and ghrelin levels, were measured.

**Results:**

Free fatty acid (FFA) levels had the most pronounced changes: IR patients had a 9-fold increase in FFA levels at day 1, and patients without IR had a 6-fold increase. Leptin levels at days 1 and 12, in IR patients were, on average, 1.5- and 2-fold higher compared to the controls and patients with no IR (р < 0.05). Leptin levels in IR patients were increased throughout the entire hospital stay. Resistin levels in IR patients were, on average, 1.4-fold higher throughout the entire hospital stay, while in non-IR patients, resistin levels were similar to the controls. Adiponectin levels in IR patients were decreased compared to the controls, while in patients with IR, they were similar to the controls. Both IR and non-IR MI patients had 3-fold and 3.7-fold lower ghrelin levels at day 1, respectively, compared to the controls. The correlation analysis showed a negative correlation between ghrelin and FFA (r = −0.48 р = 0.007), ghrelin and leptin (r = −0.4 р = 0.003), ghrelin and insulin (r = −0.54 р = 0.002), and ghrelin and glucose (r = −0.31 р = 0.002) in MI patients.

**Conclusion:**

Dyslipidaemia, along with insulinaemia and glycaemia, is one of the most significant IR risk factors in the acute and early recovery phases of MI. Dyslipidaemia is characterised by a high FFA level; an imbalance of leptin, resistin, and adiponectin; and a deficiency of ghrelin in the acute and early recovery periods of MI. FFA and ghrelin can be used as promising molecular markers to stratify the risk of recurrent acute coronary events and diabetes mellitus in MI patients.

## Background

Insulin resistance (IR) is a risk factor for ischaemic heart disease and myocardial infarction (MI) [1]. IR often manifests in MI and is regarded as an independent predictor of in-hospital mortality, which can provide early risk stratification for recurrent acute coronary events [1,2]. Currently, there is no common understanding of pathogenetic associations between IR and complicated MI. In terms of pathogenesis, IR is a rather heterogenic phenomenon; therefore, a range of parameters, including traditional hyperinsulinaemia and hyperglycaemia, are considered to be IR markers [3]. However, some lipid metabolism parameters are also thought to be promising IR markers [4], with their role in cardiovascular diseases being well established. It is known that free fatty acids (FFAs) block glucose transport by inhibiting insulin’s interaction with hepatocytes and monocytes, leading to hyperglycaemia and IR development [5]. Additionally, the current hypothesis that a range of adipose tissue and gastric endocrine cell mediators can play an important role in lipid metabolism regulation and IR development is being actively discussed [6-8]. Medical literature indicates that adipokines such as leptin, resistin and adiponectin take part in insulin production regulation [6,9]. Some existing data on the important role of ghrelin in glucose and lipid metabolism as well as energy homeostasis regulation suggest that ghrelin plays a role in IR development [7]. Despite extensive study of a wide range of IR parameters, searching for and implementation of new approaches to IR assessment seems to be relevant to predicting MI and its complications.

This study was aimed at determining the most informative lipid metabolism and adipokine status parameters to assess IR in MI.

## Methods

### Study subjects and design

The study enrolled 200 patients (130 males and 70 females aged 61.4 ± 1.12 years) diagnosed with ST elevation MI. The diagnosis was verified based on clinical, electrocardiographic (ECG), echocardiographic (ECHO) and biochemical characteristics of MI (2007 National Cardiology Society Guidelines). The exclusion criteria were a history of type 2 diabetes mellitus and severe concomitant diseases affecting the prognosis, i.e., anaemia, renal or hepatic insufficiency, cancers, worsening of infectious or inflammatory diseases, and autoimmune conditions. The inclusion criteria were MI within 24 hours from the onset and no age restrictions.

All study was carried out in compliance with the Helsinki Declaration, and its protocol was approved by the Ethical Committee of Research Institute for Complex Issues of Cardiovascular Diseases under the Siberian Branch of the Russian Academy of Medical Sciences and all patients participated the study under their written informed consent.

A structured mathematical model was used to assess IR levels at days 1and 12 based on fasting plasma insulin and glucose values and calculating the QUICKI index (Quantitative Insulin Sensitivity Check Index) (Katz A. et al. 2000) according to the following formula: QUICKI = 1/[log (I0) + log (G0)], where I0 is baseline insulinaemia (mMU/mL), and G0 is baseline glycaemia (mg/dL). According to A. Katz et al. (2000), the mean QUICKI index of 0.382 ± 0.007 correlates with normal tissue sensitivity to insulin, while the QUICKI indexes of 0.331 ± 0.010 and 0.304 ± 0.007 correlate with moderate and intensive IR [3]. According to the QUICKI index, all of the patients were divided into two groups: Group 1 included 46 patients (23%) with normal tissue sensitivity to insulin, and group 2 included 154 patients (77%) with the QUICKI index correlating to moderate and intensive IR. Clinical characteristics of the patients are presented in Table 1.

**Table 1:** Initial clinical and anamnestic characteristics of patients, n (%)

**Variable**	**Сontrol (n = 33)**	**Patients with MI without IR (n = 46)**	**Patients with MI and IR (n = 154)**	**p-value**
QUICKI, n(%)		0.380	0.308	0.025
		(0.378;0,384) (23%)	(0.306;0.10) (77%)	
Men, n(%)	18(60)	29 (63.00)	90 (58.41)	0.208
Age, (years) **Clinical measures**	58(51;62)	58.15 (44.00;73.50)	59.43 (48.01;72.10)	0.070
Body mass index, (kg/m^2^)	23.6(21.3;26.5)	25.86 (23.21;30.10)	29.97 (26.91;34.55)	0.045
Overweight, kg, n(%) **Clinical measures**		14 (30.41)	117 (75.91)	0.017
Arterial hypertension, n(%)		18 (39.11)	93 (60.40)	0.030
Current smoking, n(%)	2(6.6)	23 (50.01)	75 (48.70)	0.350
Family history of IHD, n(%)	5(16)	15 (32.60)	66 (42.86)	0.045
Clinic angina to myocardial infarction, n(%)		28 (60.87)	83 (53.90)	0.540
Previous myocardial infarction, n(%)		8 (17.39)	52 (33.76)	0.042
Cerebrovascular accident/transient ischemic attack in history		5 (10.87)	9 (5.84)	0.680
**The depth of lesion**				
- Q-wave MI		34 (74.00)	130 (84.40)	0.045
- Non-Q-wave MI		12 (26.00)	24 (15.60)	0.056
**Localization of MI**				
- Posterior		9 (19.61)	60 (38.96)	0.032
- Posterior taking the right ventricle Front		3 (6.50)	9 (5.80)	0.562
**Acute heart failure (Killip)**		12 (26.00)	69 (44.81)	0.035
-I		29 (63.00)	84 (54.51)	0.032
-II		9 (19.60)	44 (28.60)	0.034
-III		6 (13.00)	25 (16.30)	0.081
-IV		2 (4.40)	1 (0.60)	0.528

The patient groups were sex- and age-matched. The IR group had a higher incidence of such cardiovascular risk factors as arterial hypertension, hypercholesterolaemia, obesity and a family history of ischaemic heart disease (IHD). Additionally, IR patients were more often diagnosed with angina pectoris and chronic heart failure (CHF). IR in MI patients was associated with a higher incidence of anterior Q-wave MI. Among their in-hospital MI complications, IR patients often had Killip II acute heart failure, arrhythmia and early postinfarct angina. Furthermore, 50% of patients in both the groups smoked.

MI patients received medical therapy according to the 2007 National Cardiology Society Guidelines. All of the patients received ASA, clopidogrel, beta blockers, ACE inhibitors and antianginal drugs, if not contraindicated; all of the medications were administered according to the standard of care, and 100% of patients received statins (Table 2).

**Table 2:** Revascularization and drug therapy during follow-up

**Therapy, n (%)**	**All patients**
	**n = 200**
β-blockers	179 (89.5)
Angiotensin-converting enzyme	164 (82)
Calcium channel blocker	139 (69.5)
Diuretics	84 (42)
Nitrates	174 (87)
Aspirin	176 (88)
Heparin	200 (100)
Clopidogrel	157 (78.5)
Statins	200 (100)
Thrombolysis	12.2 (6.1)

Primary percutaneous coronary intervention (PCI) of the infarct-related artery was used as a reperfusion therapy in 181 (90.5%) patients; systemic thrombolysis with streptokinase (1.5 mln. ME) or pharmacological treatment were administered to 12 (96%) and 7 (3.5%) patients, respectively. The control group included 33 gender- and age-matched subjects with no cardiovascular diseases.

### Assays

Blood serum and plasma were tested. The serum was separated from venous blood by centrifugation at 3000 ***g*** for 20 min and stored at − 70°C. At days 1 and 12 from the MI onset, levels serum glucose were measured with using standard Thermo Fisher Scientific test systems (Thermo Fisher Scientific Oy, Vantaa, Finland) in a Konelab 30i biochemistry analyzer (Thermo Fisher Scientific Oy), insulin and C-peptide levels were measured with BCM Diagnostics lab kits (Germany). FFA, total cholesterol (TC), triacylglycerols (TAG), low-density lipoproteins (LDL), very-low-density lipoproteins (VLDL), apolipoprotein B (apo B), high-density lipoproteins (HDL) and apolipoprotein A1 (apo A1) C-reactive protein (CRP) levels were measured at the same study time points using standard Thermo Fisher Scientific test systems (Thermo Fisher Scientific Oy, Vantaa, Finland) in a Konelab 30i biochemistry analyzer (Thermo Fisher Scientific Oy). C-peptide and insulin concentrations were measured by ELISA with BioMedica (Waterloo, NSW, Australia) and Diagnostic Systems Laboratories (Webster, TX, USA) lab kits respectively. The intra-assay coefficients of variation (CVs) for insulin Diagnostic Systems Laboratories and C-peptide ELISA were 3.8 and 4.2%, respectively, and the inter-assay CVs were 6.9 and 7.9%, respectively. Adipokine (leptin, adiponectin, resistin and ghrelin) levels were measured with the BioVendor R&D Product (Brno, Czech Republic) and intra-assay CVs were 5.9 and 6.8%. Additionally, all of the patients had their postprandial glycaemia, insulin and C-peptide levels measured 2 hours after eating a standard solid containing 360 kcal (protein 20 g, carbohydrate 57 g and fat 9 g).

### Statistical analysis

The statistical analysis was performed using Statistica 6.1 and SPSS 17.0 for Windows. The results were presented as the median (Me) and the 25% and 75% quartiles Мe (Q1;Q3). The statistical analyses were performed using the non-parametric Mann–Whitney test and the Wilcoxon test for skewed distributions. The exact Fisher’s test was used to analyse the difference in the frequencies in two independent groups with two-sided confidence intervals. Stepwise logistic regression and an ROC-curve (receiver operating characteristic) with the area under the curve (AUC) measurement were used to determine the most informative IR parameters, the hazard ratio (HR) and the confidence interval (95%).

## Results

The IR group had increased glucose levels from day 1 to day 12. The prediabetic status in MI manifested as 1.5-, 1.4- and 1.9-fold higher postprandial glucose, insulin and C-peptide levels, respectively, compared to the controls (Table 3). IR was diagnosed in 77% of the MI patients. The MI patients without IR had mildly increased glucose levels at days 1 and 12 with normal insulin and C-peptide levels (Table 3).

**Table 3:** Basal and postprandial level glucose, insulin и C-peptide at 1 and 12 days of the myocardial infarction development

**Variable**	**Control (n = 33)**		**Patients with MI without IR (n = 46)**			**Patients with MI and IR (n = 154)**		
	**Basal level**	**Postprandial level**	**1-th day**	**12-th day**		**1-th day**	**12-th day**	
				**Basal level**	**Postprandial level**		**Basal level**	**Postprandial level**
Glucose, mmol/liter	4.40 (3.60;5.50)	4.38 (3.40;5.80)	6.20 (5.51;8.10)^*a*^	5.60 (5.01;6.20)^*b*^	5.80 (4.91;6.80)	6.70 (5.81;8.10)^*a*^	6.00 (4.80;7.01)	6.70 (5.81;8.11)^*c*^
Insulin, mU/ml	12.50 (8.70;18.50)	28.12 (4.80;43.20)	12.71 (2.51;19.80)	12.52 (2.61;15.23)	26.72 (2.37;41.01)	14.11 (8.61;21.0)	15.32 (10.52;22.50)	38.55 (4.72;63.60)^*a*^^*c*^^*d*^
C-peptide, ng/ml	1.20 (0.73;1.87)	1.78 (0.73;1.98)	1.03 (0.35;1.79)	1.01 (0.50;1.41)	2.43 (0.81;3.43)	1.01 (0.68;1.46)	1.75 (0.72;1.95)^*b*^^*c*^	3.51 (1.06;6.74)^*a*^^*d*^

Notes: ^*a*^compared with control, (p < 0.05).

^*b*^reliable differences in the parameters on the 1st and 12th (p < 0.05).

^*c*^reliable differences between the groups on day 1 (p < 0.05).

^*d*^reliable differences between the groups on day 12 (p < 0.05).

The IR group had a statistically significant increase in TC, TAG, LDL, apo-B and the apo-B/apo-A1 ratio as well as a decrease in HDL and Apo-A compared to the non-IR and control groups (Table 4). FFA levels had the most pronounced changes: IR patients had a 9-fold increase in FFA levels at day 1, and patients without IR had a 6-fold increase; the study groups were significantly different for this parameter (Table 4). By day 12, both groups had lower FFA levels relative to day 1; however, they were still more than 2-fold higher than the controls.

**Table 4:** Lipid profile in patients with myocardial infarction at 1 and 12 days of the myocardial infarction development

**Variable**	**Control, n = 33**	**Patients with MI without IR, n = 46**		**Patients with MI and IR, n = 154**	
		**1-th day**	**12-th day**	**1-th day**	**12-th day**
TC, mmol/liter	4.30 (3.51;6.10)	4.71 (3.91;6.45)	4.35 (3.90;5.00)	6.00 (5.21;7.01)^*a*^^*c*^	5.80 (4.90;7.01)^*c*^
TG, mmol/liter	1.13 (0.78;1.23)	1.15 (0.88;1.35)	1.83 (1.37;2.28)^*b*^	1.85 (1.31;2.51)^*a*^^*c*^	2.14 (1.48;2.97)^*b*^
HDL-C, mmol/liter	1.31 (1.02;1.72)	1.02 (0.82;1.17)	1.02 (0.83;1.24)	0.98 (0.74;1.30)^*a*^	0.96 (0.80;1.13)^*a*^
LDL-C, mmol/liter	2.03 (1.51;2.55)	2.13 (1.53;2.57)	2.45 (1.81;3.90)	3.10 (2.49;3.63)^*a*^^*c*^	3.35 (2.64;4.26)^*bc*^
VLDL-C, mmol/liter	0.44 (0.33;0.53)	0.52 (0.40;0.61)	0.83 (0.62;1.04)^*b*^	0.84 (0.59;1.14)^*a*^^*c*^	0.97 (0.67;1.35)^*b*^
apo B, g/L	1.02 (0.76;1.25)	1.00 (0.78;1.26)	1.28 (1.05;1.38)^*b*^	1.26 (1.07;1.51)^*a*^^*c*^	1.42 (1.04;1.69)^*b*^
apo A1, g/L	1.43 (1.29;1.73)	1.05 (0.93;1.49)^*a*^	1.54 (1.19;1.75)^*b*^	1.27 (1.15;1.43)^*a*^	1.28 (1.14;1.51)
apo-В/apo-А1	0.71 (0.59;1.01)	0.90 (0.72;1.16)	0.81 (0.69;1.09)	0.97 (0.81;1.25)^*a*^	0.94 (0.77;1.31)^*c*^
FFA, mcmol/L	0.20 (0.10;1.10)	1.20 (0.81;1.86)^*a*^	0.50 (0.43;0.69)^*b*^	1.80 (1.42;213)^*a*^^*c*^	0.57 (041;091)^*b*^

*Abbreviations*: *TC*, Total cholesterol; *TG*, Triglycerides; *HDL-C*, Cholesterol high-density lipoproteins; *LDL-C*, Cholesterol low-density lipoproteins; *VLDL-C*, Cholesterol very-low-density lipoproteins; *apo B*, Apolipoprotein B; *apo A1*, Apolipoprotein A.

Notes: ^*a*^reliable differences from control (p<0.05).

^*c*^reliable differences in the indicators on the 1 and 12days (р<0.05);

^*b*^reliable differences in the parameters between the groups (р<0.05).

At days 1 and 12, serum leptin levels in IR patients were, on average, 1.5- and 2-fold higher compared to the controls and patients with no IR (р < 0.05), respectively (Table 5). Leptin levels in IR patients were increased throughout the entire hospital stay; however, in non-IR patients, leptin levels decreased by day 12.

**Table 5:** Dynamics of adipokines and ghrelin concentrations in patients at 1 and 12 days of the myocardial infarction development

**Variable**	**Control, n = 33**	**Patients with MI without IR,**		**Patients with MI and IR,**	
		**n = 46**		**n = 154**	
		**1-th day**	**12-th day**	**1-th day**	**12-th day**
Leptin, ng/ml	6.98 (4.50;9.75)	108.2 (8.01;25.91)^*a*^	9.20 (7.27;13.0)^*b*^	15.44 (8.62;30.07)^*a*^	15.62 (8.7;25.29)^*d*^
Resistin, ng/ml	7.00 (3.81;9.82)	6.64 (3.82;11.15)	6.86 (3.74;10.51)	9.94 (8.48;10.78)^*a*^^*c*^	9.19 (7.98;12.08)^*d*^
Adiponectin, mg/ml	11.35 (7.30;13.51)	11.35 (9.71;16.56)	12.20 (9.81;17.01)	9.88 (8.41;15.20)^*a*^^*c*^	9.90 (8.34;14.71)^*d*^
Ghrelin, ng/ml	55.20 (31.61;90.21)	18.36 (18.14;18.43)^*a*^	18.21 (17.89;18.73)	15.07 (13.81;17.59)^*a*^^*c*^	14.97 (13.57;17.61)^*d*^
CRP, mg/l	1,15 (0,86:2,1)	22,6 (14,0:39,0)^*a*^	6,0 (5,0:12,0)^*b*^	27,93 (11,41:51,28)^*a*^	13,0 (4,05:22,2)^*ab*^

Notes: ^*a*^compared with control (p<0.05).

^*b*^reliable differences in the parameters on the 1 and 12days (p<0.05).

^*c*^reliable differences between the groups on day 1 (p<0.05).

^*d*^reliable differences between the groups on day 12 (p<0.05).

Resistin levels in IR patients were, on average, 1.4-fold higher throughout the entire hospital stay, while in non-IR patients, resistin levels were similar to the controls.

Adiponectin levels in IR patients were decreased compared to the controls, while in patients with preserved tissue sensitivity to insulin, they were similar to the controls.

The study showed that ghrelin levels decreased significantly unlike the other parameters under study. Both IR and non-IR MI patients had 3-fold and 3.7-fold lower ghrelin levels at day 1, respectively, compared to the controls. The MI groups were significantly different in terms of the changes in this parameter. The correlation analysis showed a negative correlation between ghrelin and FFA levels (r = −0.48 р = 0.007), ghrelin and leptin levels (r = −0.4 р = 0.003), ghrelin and insulin levels (r = −0.54 р = 0.002), and ghrelin and glucose levels (r = −0.31 р = 0.002) in MI patients.

The most informative IR markers among glucose metabolism parameters were glucose, insulin and C-peptide levels assessed at day 12 from the MI onset. Higher glucose, C-peptide and insulin levels caused 2.8-fold, 2-fold and 3.65-fold increased risks of IR, respectively. Among lipid metabolism parameters, only FFA levels were independently associated with IR development in MI. An FFA increase of 1 mmol/L at day 1 was associated with 2.9-fold increased risk of IR. However, this parameter was not highly specific and sensitive, which was shown by an AUC of 0.7, reflecting the average quality of the model (Table 5). We therefore studied diagnostic sensitivity of FFAs combined with other markers. If FFA levels were combined with another markers, the AUC increased up to 0.93, reflecting an excellent quality of the mathematical model. Measuring the key inflammation marker showed that at day 1 MI patients without IR (the whole study population) had 19,1-fold increased CRP concentrations. At the same time the patients with MI and IR CRP concentrations 24.3-fold higher (Table 5). At day 12 CRP concentrations decreased in the both groups but were still, 5.2-fold higher with the MI patients and 11.3 patients with MI and IR.

Such parameters as adiponectin, leptin and resistin were not diagnostically significant enough to detect IR in MI patients (Table 6). The AUC showed that ghrelin was the most sensitive and specific IR marker, especially in early MI phases. Low ghrelin levels at day 1 from the MI onset were associated with the risk of IR increased by 78%. Assessing ghrelin levels in combination with FFA levels increased the diagnostic significance of the latter in reference to IR detection. When these parameters were combined and assessed during the early phase of MI, AUC was 0.86, which reflected the high quality of the model.

**Table 6:** The odds ratio (OR) for the development of insulin resistance in the hospital stay of myocardial infarction

**Variable**	**1-th day MI**				**12-th day MI**			
	**OR**	**95%-й CI**	**p-value**	**AUG**	**OR**	**95%-й CI**	**p-value**	**AUG**
Glucose, mmol/liter	1.17	0.97-1.40	0.090	0.58	2.80	1.66-4.73	0.000	0.79
Insulin, mU/ml	1.10	1.04-1,17	0.002	0.76	3.65	1.79-7.41	0.000	0.98
C-peptide, ng/ml	1.08	0.72-1.62	0.710	0.58	2.10	1.02-4.33	0.040	0.64
FFA, mcmol/L	2.90	1.38-6.11	0.005	0.70	1.82	0.60-5.51	0.290	0.56
Adiponectin, mg/ml	0.94	0.88-1.01	0.110	0.61	0.97	0.91-1.02	0.230	0.63
Leptin, ng/ml	1.01	0.98-1.05	0.380	0.57	1.06	1.01-1.11	0.020	0.66
Resistin,ng/ml	0.96	0.90-1.03	0.240	0.66	0.91	0.85-0.99	0.120	0.70
Ghrelin, ng/ml	0.22	0.09-0.52	0.000	0.87	0.57	0.42-0.76	0.000	0.82

## Discussion

Studying mechanisms of IR development in different pathologic conditions, including MI, is a relevant issue. According to P J Stubbs et al. (1999), IR in MI patients can help predict adverse disease outcome within 3 years of follow-up [10,11]. IR manifestation in the early phase of MI is one of the typical body responses to catecholamine stress. In this study, new IR was diagnosed in 77% of MI patients during their hospital stay (Table 1) and was associated with a more severe disease course: higher incidences of large Q-wave MI and in-hospital complications.

In this study, IR was diagnosed using traditional markers, i.e., serum insulin and glucose levels as well as the IR index [3]. Basal and postprandial hyperglycaemia, high postprandial insulin and C-peptide levels in the early recovery phase, and decreased QUICKI index were the markers of IR. The increase in glucose, insulin and C-peptide levels were associated with 2.8-, 3.65- and 2-fold higher risks of IR, respectively (Table 5). This can be regarded as a consequence of pancreatic dysfunction and impaired β-adrenergic glucose metabolism regulation in hepatocytes during catecholamine stress.

IR in MI patients was associated with a cluster of cardiovascular risk factors, including hypertension, obesity and dyslipidaemia, characterised by higher VLDL and TAG levels and lower HDL levels (Table 3). Moreover, IR patients had a sharp rise in plasma FFA, which might be a marker of excess lypolysis activation, impaired energy homeostasis in cardiomyocytes and IR manifestation in MI. This assumption is supported by the results of the logistic regression analysis: among all of the lipid profile markers, only FFA levels were closely associated with IR in MI (Table 5). Higher FFA levels in the acute phase of the disease were associated with a 3-fold increased IR risk. Measuring both FFA and insulin levels in the acute phase led to a better diagnostic value of FFAs, which is logical in terms of pathogenesis. FFAs are able to stimulate insulin secretion in pancreatic beta-cells, decrease insulin hepatic clearance, and impair receptor and postreceptor insulin signalling, which all result in postprandial hyperinsulinaemia and IR progression [12].

Adipokines, which are the essential regulators of energy metabolism that modulate insulin synthesis and secretion, play an important role in IR pathogenesis [13,14]. To study the role of adipokine status, we chose markers with different mechanisms of action towards insulin: leptin and resistin are IR mediators and inductors, while adiponectin increases tissue sensitivity to insulin [14]. Low adiponectin levels observed in diabetes, metabolic syndrome and coronary artery disease can lead to IR [14].

The study results showed the increased adipokine levels, which intensified IR (Table 4). High leptin and resistin levels were accompanied by a higher IR index during the entire hospital stay. Leptin in supraphysiological doses in vitro is known to block insulin interaction with its receptor on the cell membrane, which is associated with impaired insulin-mediated glucose transport, hyperglycaemia and more severe IR [15,16]. Additionally, according to Opie et. al. (2008), high leptin levels intensify FFA oxidation and lead to diacylglyceride build-up, which, in turn, induces IR [17]. Another adipokine, resistin, is an antagonist of insulin [18]. Resistin inhibits insulin-mediated glucose uptake by the target tissues and decreases FFA consumption and their metabolism in the skeletal muscles through the activation of AMP-activated protein kinase [19]. Generally, the increase in the above-mentioned adipokine levels can have a negative effect on insulin production, secretion and cell signalling, which may induce IR in MI.

Unlike leptin and resistin, the protective effects of adiponectin decreased during the hospital stay, especially in IR patients. Adiponectin is known to neutralise the lipotoxic effect of FFA, inducing endothelial dysfunction and IR [20]. The decrease in adiponectin levels is likely to promote FFA lipotoxic effect, which certainly contributes to IR development and progression. This assumption is supported by the results of a correlation analysis showing a negative correlation between FFA and adiponectin levels.

The role of ghrelin, a gastrointestinal endocrine peptide and an important regulator of growth factor secretion, desire for food and energy homeostasis, in IR pathogenesis has been actively discussed recently [7,21,22]. It was found that cardiomyocytes are able to produce ghrelin, which has diverse protective effects, including the inhibition of cardiomyocyte and endothelial cell apoptosis and improved left ventricular function in ischaemia/reperfusion [23].

Ghrelin is known to modulate insulin secretion and, therefore, is regarded as a promising molecular IR marker. Ghrelin was shown to contribute to the expression of α- and β-insulin receptor subunits. At the same time, 1–10 nm/L of insulin inhibit basal and noradrenaline-stimulated ghrelin secretion but do not influence ghrelin mRNA expression [24]. Obese children have lower ghrelin levels that those with normal metabolism; at the same time, ghrelin had a strong positive correlation with the HOMA-IR index irrespective of anthropometric and metabolic parameters of IR syndrome [25]. Additionally, in diabetic patients, metformin therapy, which improves tissue sensitivity to insulin, was accompanied by higher ghrelin levels [26-28]. In this study, ghrelin levels were significantly decreased in MI patients during the entire hospital stay; in patients with IR, the changes were more pronounced. Previously, it was shown that MI patients have decreased ghrelin levels, which, in the authors’ opinion, is due to an enhanced binding of ghrelin with its receptor in ischaemia/reperfusion [29]. We suggest that in MI, the inhibition of ghrelin secretion may also be due to the imbalance in the adipokine system accompanied by the dysfunction of insulin-secreting pancreatic cells, impaired lipid metabolism and IR manifestation. Our assumption is supported by the results of experimental studies that demonstrate the antagonistic relationship between leptin and ghrelin [30,31] and the ability of high FFA levels to block ghrelin secretion [7] as well as by the result of a correlation analysis showing the negative correlation between leptin and ghrelin and between insulin and FFA in MI patients. Additionally, the ghrelin level appears to be a more informative IR marker than traditional markers and adipokine status parameters in both the acute and recovery MI phases (Table 5). Ghrelin, as a marker of IR in MI, has high diagnostic sensitivity and specificity (82-87%). Ghrelin has a better diagnostic value than insulin in the acute MI phase, and assessing both ghrelin and FFA levels increased the diagnostic significance of the latter with regard to IR.

## Conclusion

Thus, insulin resistance was observed in 77% of MI patients and was associated with a history of cardiovascular risk factors, an adverse disease course and impaired lipid metabolism. Among lipid metabolism parameters, FFAs are of the greatest interest in terms of IR detection in MI patients. The 9-fold higher FFA levels in the acute MI phase are associated with a 3-fold higher risk of impaired tissue sensitivity to insulin. The diagnostic value of FFA assessment is higher when insulin and ghrelin levels are also measured. Adipokine imbalance in MI manifesting in higher leptin and resistin levels, which impair tissue sensitivity to insulin and inhibit protective adiponectin effects, is accompanied by IR development. Ghrelin levels are the most informative marker in terms of IR detection in the acute phase of MI; it has high specificity and sensitivity. A 4-fold decrease in ghrelin levels is associated with a 78%-higher risk of IR.

## Competing interests

The authors declare that they have no competing interests.

## Authors’ contributions

OG was principal investigator, study co-ordinator and investigator, participated in all stages of recruitment of the patients and in analysis of the data, drafted and reviewed critically the manuscript. EU was study co-ordinator and investigator, participated in all stages of recruitment of the patients and in analysis of the data, drafted and reviewed critically the manuscript; EB and YD was study investigator, participated in all stages of recruitment of patients and reviewed critically the manuscript. OB was principal investigator. All other study investigators conducted the study and collected the data. All authors read and approved the final manuscript.

## Pre-publication history

The pre-publication history for this paper can be accessed here:

http://www.biomedcentral.com/1471-2261/14/7/prepub
